# Mst1/Hippo signaling pathway drives isoproterenol-induced inflammatory heart remodeling

**DOI:** 10.7150/ijms.95850

**Published:** 2024-07-01

**Authors:** Xiuling He, Shuai Huang, Chijia Yu, Ye Chen, Hang Zhu, Jianwei Li, Shanshan Chen

**Affiliations:** 1 Department of Cardiology, School of Medicine, South China University of Technology, Guangzhou, 510006, China.; 2 Department of Cardiology, The Sixth Medical Center of PLA General Hospital of Beijing, Beijing, 100048, China.; 3 Department of Cardio-Thoracic Surgery, The Third Affiliated Hospital, Sun Yat-Sen University, 510630, Guangzhou, China; 4 Department of Clinical Laboratory Medicine, The First Medical Centre, Medical School of Chinese PLA, Beijing, China.; 5 Department of Critical Care Medicine, Zhongshan City People's Hospital, Zhongshan 528403, China.

**Keywords:** Mst1, isoproterenol, myocardial injury, inflammation response, cardiac remodeling

## Abstract

Isoproterenol (ISO) administration is a well-established model for inducing myocardial injury, replicating key features of human myocardial infarction (MI). The ensuing inflammatory response plays a pivotal role in the progression of adverse cardiac remodeling, characterized by myocardial dysfunction, fibrosis, and hypertrophy. The Mst1/Hippo signaling pathway, a critical regulator of cellular processes, has emerged as a potential therapeutic target in cardiovascular diseases. This study investigates the role of Mst1 in ISO-induced myocardial injury and explores its underlying mechanisms. Our findings demonstrate that Mst1 ablation in cardiomyocytes attenuates ISO-induced cardiac dysfunction, preserving cardiomyocyte viability and function. Mechanistically, Mst1 deletion inhibits cardiomyocyte apoptosis, oxidative stress, and calcium overload, key contributors to myocardial injury. Furthermore, Mst1 ablation mitigates endoplasmic reticulum (ER) stress and mitochondrial fission, both of which are implicated in ISO-mediated cardiac damage. Additionally, Mst1 plays a crucial role in modulating the inflammatory response following ISO treatment, as its deletion suppresses pro-inflammatory cytokine expression and neutrophil infiltration. To further investigate the molecular mechanisms underlying ISO-induced myocardial injury, we conducted a bioinformatics analysis using the GSE207581 dataset. GO and KEGG pathway enrichment analyses revealed significant enrichment of genes associated with DNA damage response, DNA repair, protein ubiquitination, chromatin organization, autophagy, cell cycle, mTOR signaling, FoxO signaling, ubiquitin-mediated proteolysis, and nucleocytoplasmic transport. These findings underscore the significance of Mst1 in ISO-induced myocardial injury and highlight its potential as a therapeutic target for mitigating adverse cardiac remodeling. Further investigation into the intricate mechanisms of Mst1 signaling may pave the way for novel therapeutic interventions for myocardial infarction and heart failure.

## Introduction

Cardiovascular diseases, particularly myocardial infarction (MI) and heart failure, constitute a substantial global health burden. Isoproterenol, a synthetic catecholamine and non-selective β-adrenergic agonist, is frequently used to model MI and study cardiac remodeling mechanisms 1. T This model replicates key features of human MI, including myocardial necrosis, inflammation, and subsequent detrimental remodeling. Isoproterenol-induced cardiac damage triggers a complex inflammatory cascade, central to the progression of adverse remodeling 2-4. The initial insult elicits the release of damage-associated molecular patterns (DAMPs) from necrotic cardiomyocytes, activating pattern recognition receptors (PRRs) on immune and resident cardiac cells 5, 6. This activation orchestrates the recruitment and infiltration of diverse inflammatory cells, such as neutrophils, monocytes, and lymphocytes, into the myocardium 7. Neutrophils, early responders to injury, release reactive oxygen species and proteolytic enzymes, exacerbating tissue damage. Subsequently recruited monocytes differentiate into pro-inflammatory M1 macrophages, sustaining the inflammatory response through cytokine and chemokine secretion 8, 9. This persistent inflammatory milieu drives adverse remodeling, characterized by cardiomyocyte hypertrophy, interstitial fibrosis, and ultimately, impaired cardiac function 9. While isoproterenol's utility in modeling MI is evident, the precise molecular mechanisms governing inflammation-induced cardiac remodeling remain incompletely understood. Elucidating the intricate interactions between inflammatory and resident cardiac cells, along with their mediators, may pave the way for targeted therapies aimed at mitigating adverse remodeling and improving outcomes in patients with MI and heart failure.

The evolutionarily conserved Mst1/Hippo signaling cascade is a key regulator of fundamental cellular processes such as proliferation, apoptosis, and organ size 10. Recent investigations have revealed its pivotal role in cardiovascular physiology and disease, highlighting potential therapeutic targets for a spectrum of cardiac and vascular pathologies. Central to this pathway is a kinase cascade initiated by mammalian sterile 20-like kinases (Mst1/2), culminating in the phosphorylation and activation of large tumor suppressor kinases (Lats1/2) 11. Activated Lats kinases subsequently suppress the transcriptional co-activators YAP (Yes-associated protein) and TAZ (transcriptional co-activator with PDZ-binding motif), thereby modulating gene expression programs essential for cardiovascular homeostasis 12.

In the heart, the Mst1/Hippo pathway orchestrates cardiomyocyte proliferation, survival, and growth 13. During embryonic development, it constrains cardiomyocyte proliferation to ensure proper cardiac size and function 14. Conversely, in the adult heart, perturbations in this pathway contribute to maladaptive remodeling and heart failure 15. Notably, Mst1 activation and subsequent YAP/TAZ inhibition have been implicated in cardiomyocyte death, pathological growth, and fibrosis, thereby exacerbating cardiac dysfunction in diverse disease models 16, 17.

This study aims to elucidate the role of the Mst1/Hippo pathway in cardiac remodeling, with a particular emphasis on isoproterenol-induced myocardial injury.

## Methods

### Ethical statement

This study adhered to the Declaration of Helsinki and the ethical guidelines of South China University of Technology. The Ethics Committee approved the experimental protocols, with the reference number SCUT-2023ST.

### Animals

Cardiac-specific *Mst1* knockout (*Mst1^Cko^*) mice were generated by crossing *Mst1* floxed mice with α-myosin heavy chain Cre transgenic mice (*α-MHC^Cre+^*), as previously described 18. To induce myocardial injury, mice were administered isoproterenol (100 mg/kg) for two consecutive days. Cardiac tissues were harvested four weeks post-treatment for functional analyses.

### Echocardiography

Transthoracic echocardiography was performed on anesthetized mice (0.5-1% isoflurane, heart rate maintained at ~550 bpm) using a Vevo2100 system equipped with an MS-550D 40-MHz probe (VisualSonics). Left ventricular (LV) systolic and diastolic dimensions were measured in M-mode along the parasternal short axis. Fractional shortening (FS) was calculated as: FS (%) = 100 × (LV end-diastolic diameter - LV end-systolic diameter) / (LV end-diastolic diameter) 19.

### Assessment of total superoxide production

Superoxide production within freshly excised murine hearts was assessed using dihydroethidium (DHE) fluorescent imaging. Hearts were initially perfused via the pulmonary artery with Krebs HEPES buffer (KHB) to remove blood, then embedded in OCT compound and cryosectioned (5 μm) at -20°C. Sections were washed with KHB and incubated with 2 μmol/L DHE (MilliporeSigma) in a light-protected, humidified chamber at 37°C for 30 minutes. After rinsing to remove excess DHE, fluorescent images indicative of total superoxide levels were immediately acquired using an Olympus BX51 microscope (excitation 520 nm, emission 610 nm).

### Immunofluorescence staining

Samples were fixed in paraformaldehyde, followed by washing with PBS. Antigen retrieval was performed by heating in 10 mM sodium citrate buffer (pH 6.0) with 0.05% Tween 20 for 12 minutes at 95°C. Slides were then washed again with PBS and incubated with Dako Protein Block Serum-free (containing 10% normal goat serum) for 1 hour at room temperature to minimize non-specific antibody binding. Primary antibody incubation was carried out overnight at 4°C. Following washes with PBST (PBS containing 0.2% Tween 20) and PBS, slides were incubated with the appropriate Alexa Fluor-conjugated secondary antibodies (1:2000, Invitrogen) and DAPI (2.5 ug/mL, Invitrogen) for 1 hour at room temperature. After final washes with PBST and PBS, coverslips were mounted and immunofluorescence images were acquired using an Olympus FX1200 MPE confocal laser scanning microscope.

### Cell culture and transfection

HL-1 cells were seeded in 6-well plates and cultured in DMEM supplemented with 10% FBS. Mst1-targeting siRNA (Santa Cruz Biotechnology) was delivered using Lipofectamine 2000 in DMEM. Transfection efficiency was confirmed 48 hours later by visualizing GFP expression under a Zeiss Axio observer fluorescence microscope. For isoproterenol treatment, cells were exposed to a final concentration of 20 µM isoproterenol, then harvested for subsequent assays.

### TUNEL assay

Apoptotic cell death was evaluated using the TUNEL assay, performed with the ApoTag peroxidase *in situ* apoptosis detection kit (Millipore).

### Ca^2+^ transient-mediated myocardial contraction measurements

HL-1 cells were loaded with 5 µM Rhod-4 AM calcium indicator (AAT Bioquest) and 0.04% Pluronic F-127 (ThermoFisher) in supplemented Claycomb medium for 45 minutes at 37°C. Following removal of excess dye with PBS washes, live cells were immediately imaged in supplemented Claycomb medium containing 1.8 mM CaCl2 using a Zeiss LSM710 confocal microscope equipped with a 40x/1.20 NA C-Apochromat water-immersion objective. Images were acquired at 488 nm excitation (BIN1 channel) and 561 nm excitation (Rhod-4 AM channel), with emission collected at 495-590 nm and >570 nm, respectively. Single-frame snapshots were captured for BIN1, while time-lapse sequences (2,000 frames, 30 ms exposure) were recorded for Rhod-4 AM. Calcium transient amplitude was quantified using Fiji software. In parallel, cardiomyocyte contractility was assessed under 1 Hz field stimulation using an IonOptix system, as previously described 41,42. Briefly, contractions were elicited via platinum electrodes delivering rectangular depolarizing pulses. Cell shortening was quantified by edge detection, while calcium transients were monitored via epifluorescence after loading with 1 μmol/l Fura-2 AM (Invitrogen). Contractility and calcium data were recorded, with 5-10 steady-state contractions per cell analyzed using IonWizard software.

### Transmission electron microscopy

For perfusion fixation, mice were heparinized and euthanized by cervical dislocation. The heart was cannulated via the aorta on a Langendorff apparatus and sequentially perfused with Ca Tyrode solution (135 mM NaCl, 5.4 mM KCl, 5 mM MgCl_2_, 1 mM CaCl_2_, 0.33 mM NaH_2_PO_4_, 10 mM HEPES, pH 7.3) for 5 minutes, followed by Ca^2+^-free Tyrode solution (135 mM NaCl, 5.4 mM KCl, 5 mM MgCl_2_, 0.02 mM CaCl_2_, 0.33 mM NaH_2_PO_4_, 10 mM HEPES, pH 7.3), and finally with 2.5% glutaraldehyde in 0.15 M sodium cacodylate buffer (pH 7.4). Small (~1 mm3) sections of the left ventricle and papillary muscle were excised, post-fixed overnight at 4°C in 2% osmium tetroxide partially reduced with 0.8% K_4_Fe(CN)_6_ in 0.15 M Na-cacodylate buffer. Samples were then contrasted with 1% uranyl acetate, dehydrated in an acetone gradient, and embedded in Spurr's resin. Longitudinal ultrathin sections (65-80 nm) were cut using a Leica UCT ultramicrotome equipped with a Diatome diamond knife, collected on formvar-coated copper grids, and imaged using an FEI Tecnai 12 transmission electron microscope (TEM) fitted with an AMT XR-111 CCD camera at 3,200-15,000× magnification (80 kV).

### Real-time PCR (qRT-PCR)

Upon experiment completion, cells were rinsed with PBS and total RNA was extracted using TRIzol reagent (Invitrogen). Quantitative real-time PCR (qRT-PCR) was performed as previously detailed. Briefly, each 10 μl reaction contained cDNA derived from 20 ng of total RNA, iQ SYBR Green Supermix (Bio-Rad), and 0.5 μM of each gene-specific primer (sequences provided in Supplemental Table). Reactions were performed in triplicate using a CFX96 Touch Real-Time PCR Detection System (Bio-Rad) with the following cycling conditions: 95°C for 10 minutes, followed by 40 cycles of 95°C for 15 seconds and 60°C for 1 minute. Relative mRNA expression levels were determined using the 2-ΔΔCt method, normalizing target gene Ct values to those of reference genes. All primers were purchased from Sigma-Aldrich.

### MTT and ELISA

Cell viability was assessed using the MTT assay. Cells were seeded in a 96-well plate and allowed to attach overnight. Following treatment with the desired compounds, MTT solution (3-(4,5-dimethylthiazol-2-yl)-2,5-diphenyltetrazolium bromide) was added to each well, and the plates were incubated for 2-4 hours. Viable cells metabolized MTT into formazan crystals, which were subsequently dissolved in DMSO. Absorbance of the resulting solutions was measured at 570 nm using a microplate reader, with absorbance values directly correlating to cell viability.

Target protein levels were quantified using an ELISA. Briefly, a 96-well plate was coated with a capture antibody specific to the protein of interest. After blocking non-specific binding sites, samples and standards were added, allowing the target protein to bind to the capture antibody. Following washes to remove unbound material, an enzyme-conjugated detection antibody, also specific to the target protein, was added. After further washing, a substrate solution was introduced, reacting with the enzyme to produce a color change. The color intensity, measured spectrophotometrically, was directly proportional to the amount of target protein present. A standard curve generated with known protein concentrations enabled quantification of the protein levels in the samples.

### Western blotting analyses

Protein samples (10-40 μg) were resolved by 10% SDS-PAGE and transferred to nitrocellulose membranes. Membranes were blocked with 5% non-fat milk in PBS-Tween 20 (0.1%) for 2 hours, then probed overnight at 4°C with primary antibodies. Following incubation with species-specific HRP-conjugated secondary antibodies (Bio-Rad), signals were detected using enhanced chemiluminescence substrate (Thermo Fisher Scientific). Band intensities were quantified using NIH ImageJ software.

### Statistical analysis

Statistical analyses of data were completed using Prism software version 9.5.1. One-way ANOVA followed by Dunnett's or Turkey's multiple comparison test was applied to means of multiple experimental groups. Student's t-test was also performed for comparison of two groups. Non parametric tests (Independent-Samples Kruskal-Wallis test) were performed for data with n less than 6 using SPSS software version. A statistical probability (p) value < 0.05 was considered significant. All of the precise p values have been clearly and individually presented in the Results section and each of the figure panels.

## Results

### Mst1 ablation mitigates isoproterenol-induced myocardial dysfunction and preserves cardiomyocyte viability

**Figure 1:**
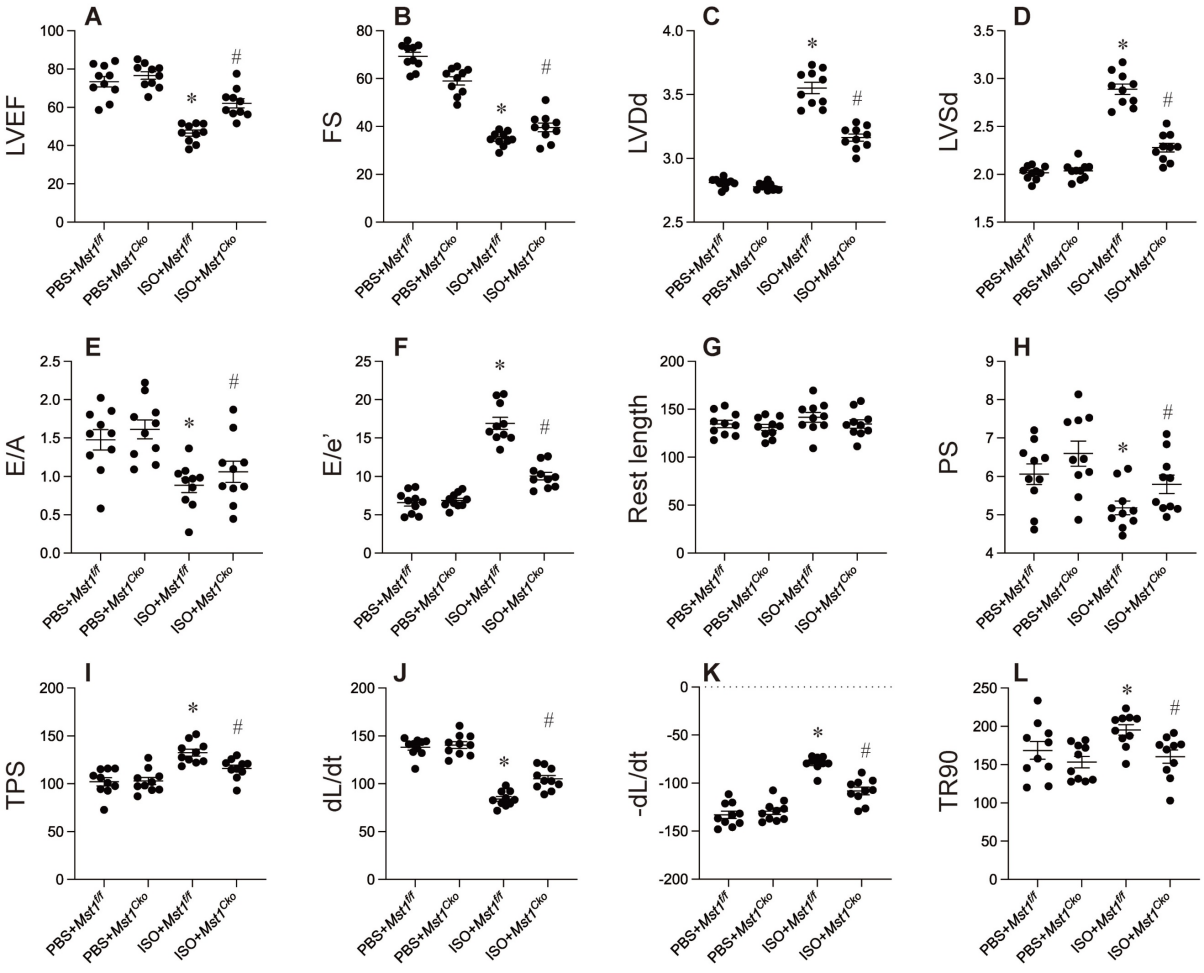
** Mst1 ablation mitigates isoproterenol-induced myocardial dysfunction and preserves cardiomyocyte viability.** Cardiac-specific *Mst1* knockout (*Mst1^Cko^*) mice and *Mst1^f/f^* mice were treated with isoproterenol (ISO, 100 mg/kg) for two consecutive days. Cardiac tissues were harvested four weeks post-treatment for functional analyses. **A-F.** Echocardiography was used to evaluate the heart function. **G-L.** Cardiomyocytes were isolated from heart and the cardiomyocyte contractility was assessed under 1 Hz field stimulation using an IonOptix system. Briefly, contractions were elicited via platinum electrodes delivering rectangular depolarizing pulses. *p<0.05 vs. PBS+* Mst1^f/f^* mice, #p<0.05 vs. ISO+* Mst1^f/f^* mice.

To elucidate the function of Mst1 in isoproterenol-induced cardiac dysfunction, we utilized cardiomyocyte-specific *Mst1* knockout (*Mst1^Cko^*) mice and their control littermates (*Mst1^f/f^*). Both groups were administered isoproterenol (100 mg/kg) for a period of three days, followed by echocardiographic assessment of cardiac performance. Isoproterenol treatment led to a significant deterioration in myocardial contractility indices (LVEF, FS, LVDs) and diastolic function parameters (E/A, E/e', LVDd) in *Mst1^f/f^* mice (Figure 1A-F). Remarkably, cardiomyocyte-specific ablation of *Mst1* maintained both systolic and diastolic function in *Mst1^Cko^* mice (Figure 1A-F). These observations were further substantiated by *ex vivo* evaluation of freshly isolated cardiomyocytes. Although isoproterenol administration did not alter resting cell length in either cohort, cardiomyocytes from isoproterenol-treated *Mst1^f/f^* mice displayed impaired contractile properties (diminished peak shortening, maximal velocity of shortening, time-to-peak shortening) and relaxation kinetics (reduced maximal velocity of relengthening, time-to-90% relengthening) (Figure 1G-L). Conversely, these parameters were ameliorated in cardiomyocytes from isoproterenol-treated *Mst1^Cko^* mice. Taken together, our findings demonstrate that *Mst1* deletion confers protection against isoproterenol-induced cardiac dysfunction and maintains cardiomyocyte functionality.

### Mst1 ablation protects against isoproterenol-induced cardiomyocyte apoptosis, oxidative stress, and calcium overload

**Figure 2:**
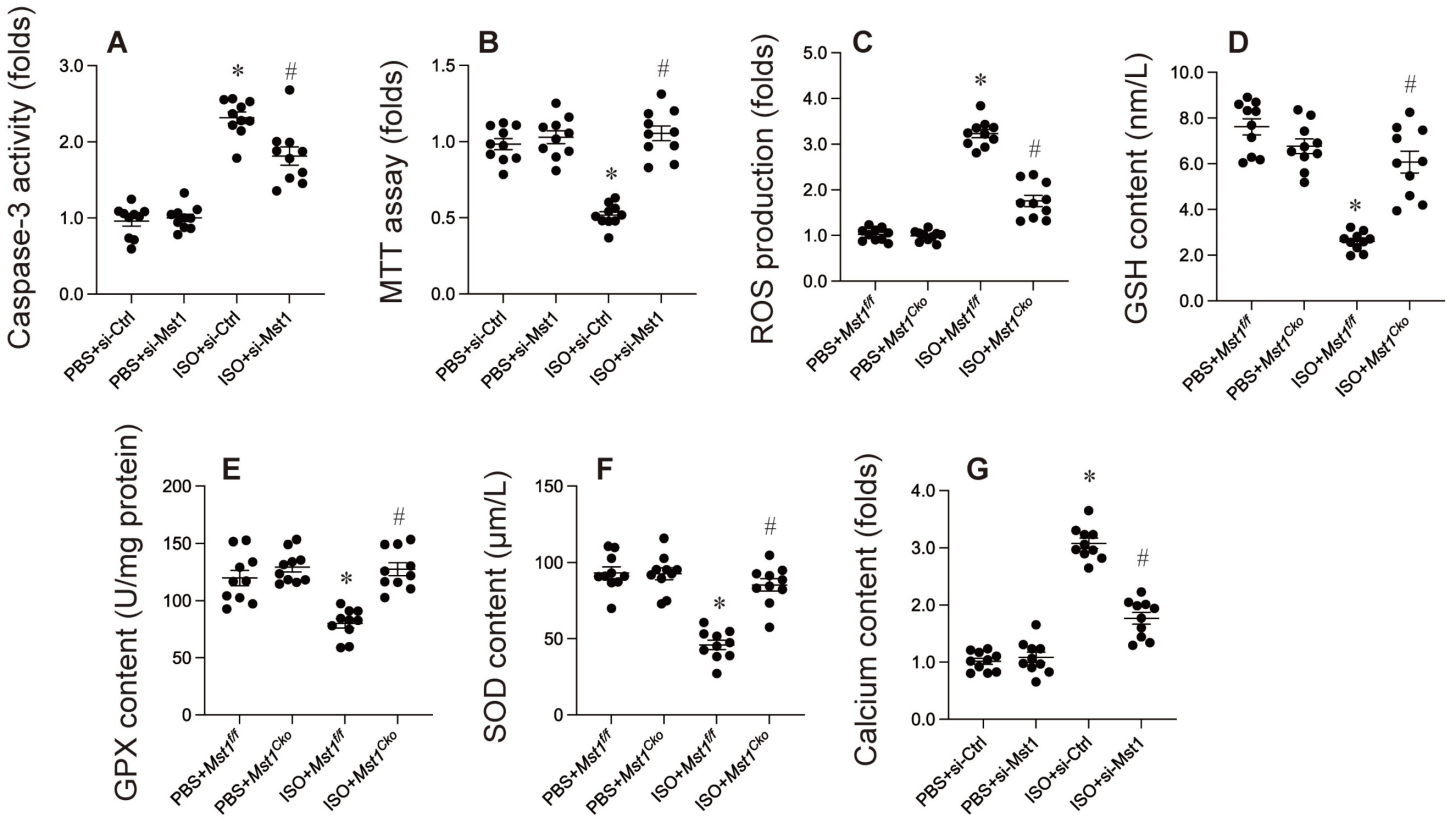
** Mst1 ablation protects against isoproterenol-induced cardiomyocyte apoptosis, oxidative stress, and calcium overload.** Cardiac-specific *Mst1* knockout (*Mst1^Cko^*) mice and *Mst1^f/f^* mice were treated with isoproterenol (ISO, 100 mg/kg) for two consecutive days. Cardiac tissues were harvested four weeks post-treatment for functional analyses. *In vitro*, HL-1 cells were transfected with Mst1-targeting siRNA (si-Mst1) or control siRNA (si-Ctrl) and then treated with 20 µM isoproterenol for 24 hrs.** A.** ELISA was used to evaluate the caspase-3 activity.** B.** Cell viability was measured by MTT assay. **C.** ROS production was determined by immunofluorescence. **D-F.** ELISA kits were used to evaluate the concentration of GSH, SOD, and GPX.** G.** Calcium content was measured by immunofluorescence in HL-1 cells. *p<0.05 vs. PBS+* Mst1^f/f^* mice, #p<0.05 vs. ISO+* Mst1^f/f^* mice.

Previous investigations have indicated an association between isoproterenol administration and cardiomyocyte apoptosis, oxidative stress, and calcium overload. In our research, HL-1 cells were exposed to isoproterenol to replicate isoproterenol-induced myocardial injury *in vitro*. HL-1 cells were transfected with siRNA targeting Mst1 and its corresponding negative control. Subsequent to this, cell apoptosis was assessed via MTT assay and caspase-3 activity measurement. As illustrated in Figure 1A, isoproterenol elevated caspase-3 activity in HL-1 cells transfected with si-Ctrl, but not in those transfected with si-Mst1 (Figure 2A). Furthermore, compared to untreated HL-1 cells, cell viability was markedly diminished following isoproterenol exposure; however, this reduction was reversed by si-Mst1 transfection (Figure 2B). Immunofluorescence analysis revealed that isoproterenol treatment enhanced ROS production in HL-1 cells, an effect that was mitigated by si-Mst1 transfection (Figure 2C). ELISA kits further demonstrated that the levels of antioxidative enzymes, including GSH, GPX, and SOD, were significantly decreased in response to isoproterenol treatment (Figure 2D-F). Notably, Mst1 knockdown preserved the levels of these antioxidative enzymes in isoproterenol-treated HL-1 cells (Figure 2D-F). Additionally, our study showed through immunofluorescence that calcium concentration was significantly increased following isoproterenol treatment, a change that was not observed in si-Mst1-transfected HL-1 cells (Figure 2G). Collectively, our findings indicate that isoproterenol induces cardiomyocyte apoptosis, oxidative stress, and calcium overload via Mst1 in HL-1 cells *in vitro*.

### Endoplasmic reticulum stress and mitochondrial fission are inhibited by Mst1 deletion in isoproterenol-mediated myocardial injury

**Figure 3:**
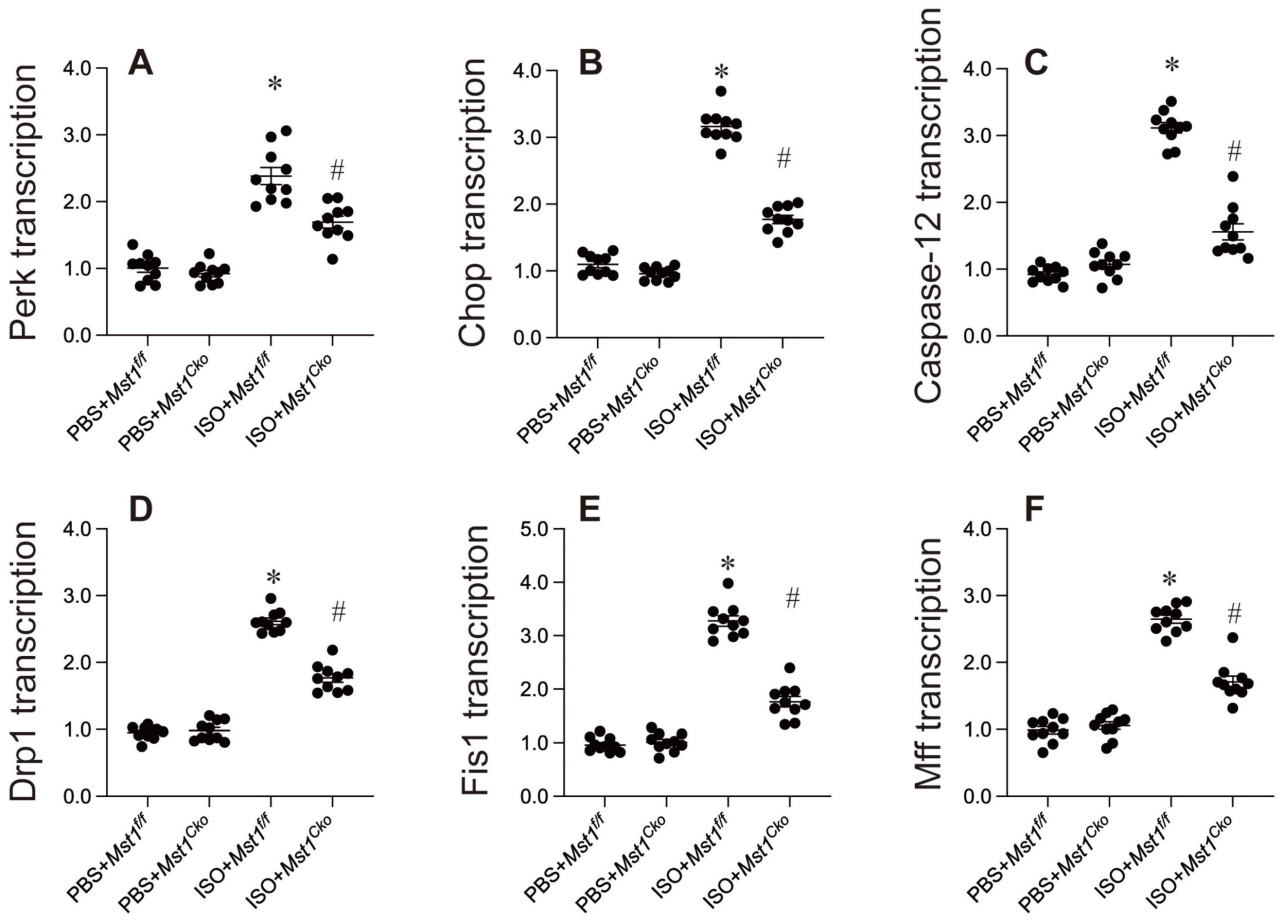
** Endoplasmic reticulum stress and mitochondrial fission are inhibited by Mst1 deletion in isoproterenol-mediated myocardial injury.** Cardiac-specific *Mst1* knockout (*Mst1^Cko^*) mice and *Mst1^f/f^* mice were treated with isoproterenol (ISO, 100 mg/kg) for two consecutive days. Cardiac tissues were harvested four weeks post-treatment for functional analyses. **A-C.** qPCR analysis of the markers of ER stress. **D-E.** RNA was isolated from heart tissues and the transcription of mitochondrial fission-related genes were determined by qPCR. *p<0.05 vs. PBS+* Mst1^f/f^* mice, #p<0.05 vs. ISO+* Mst1^f/f^* mice.

Mechanistically, endoplasmic reticulum (ER) stress and mitochondrial dysfunction have been implicated in the development of isoproterenol-induced myocardial injury. Our data support this hypothesis, revealing that isoproterenol exposure significantly increased the mRNA expression of key ER stress markers, including caspase-12, Chop, and Perk (Figure 3A-C). Importantly, cardiomyocyte-specific Mst1 deletion effectively attenuated this upregulation (Figure 3A-C), suggesting a protective role for Mst1 ablation against ER stress.

Mitochondrial fission, an early hallmark of mitochondrial dysfunction, has also been associated with myocardial injury. Isoproterenol induced a rapid increase in the mRNA levels of mitochondrial fission-related proteins such as Drp1, Fis1, and Mff, an effect that was mitigated by Mst1 deletion (Figure 3D-F).

Collectively, our results highlight the critical involvement of ER stress and mitochondrial fission in isoproterenol-induced myocardial injury. Mst1 appears to play a pivotal role in regulating these processes, as its deletion attenuated both ER stress and mitochondrial fission. These findings underscore the potential therapeutic value of targeting the Mst1 pathway in mitigating myocardial injury.

### Inflammation response is controlled by Mst1 in in isoproterenol-mediated myocardial injury

**Figure 4:**
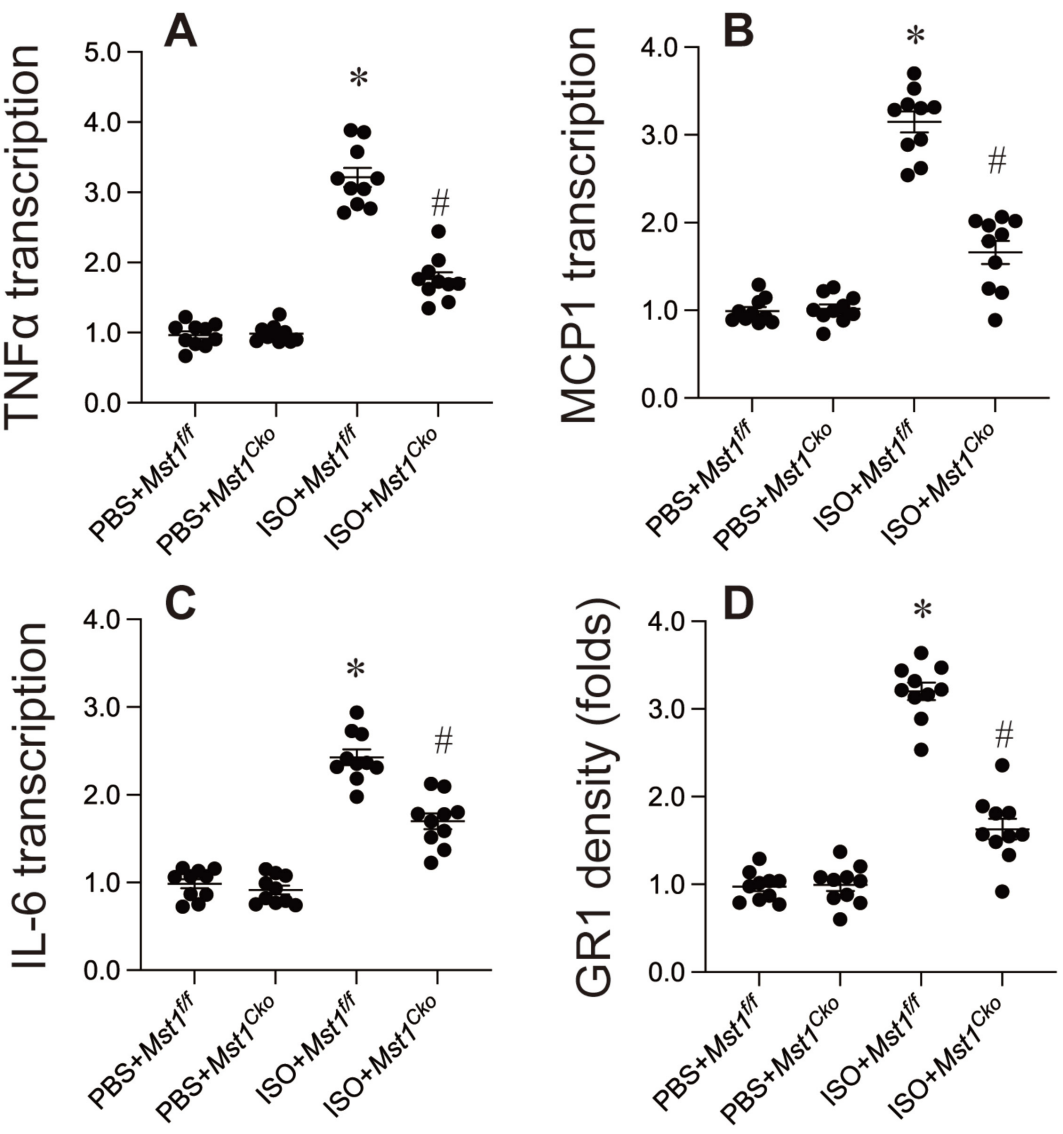
** Inflammation response is controlled by Mst1 in in isoproterenol-mediated myocardial injury.** Cardiac-specific *Mst1* knockout (*Mst1^Cko^*) mice and *Mst1^f/f^* mice were treated with isoproterenol (ISO, 100 mg/kg) for two consecutive days. Cardiac tissues were harvested four weeks post-treatment for functional analyses. **A-C.** RNA was isolated from heart tissues and the transcription of inflammation-related genes were determined by qPCR. **D.** Immunofluorescence was used to measure the levels of GR-1 in myocardium. *p<0.05 vs. PBS+* Mst1^f/f^* mice, #p<0.05 vs. ISO+* Mst1^f/f^* mice.

Inflammation is a critical factor in isoproterenol-induced cardiac remodeling. We investigated the role of Mst1 in this process and found that isoproterenol treatment significantly increased the expression of pro-inflammatory cytokines (TNFα, IL-6, MCP1) in cardiac tissue, as assessed by qPCR (Figure 4A-C). Notably, this upregulation was absent in *Mst1^Cko^* mice (Figure 4A-C), suggesting that Mst1 is required for isoproterenol-induced inflammatory gene expression. Furthermore, immunofluorescence analysis revealed that isoproterenol induced a robust infiltration of neutrophils into the myocardium, a hallmark of inflammatory response. This effect was completely abrogated in *Mst1^Cko^* mice (Figure 4D), indicating that Mst1 is essential for neutrophil recruitment in this context. Collectively, our data demonstrate that Mst1 plays a pivotal role in modulating the inflammatory response following isoproterenol-induced myocardial injury. The absence of Mst1 not only prevents the upregulation of pro-inflammatory cytokines but also inhibits neutrophil infiltration, highlighting its potential as a therapeutic target for mitigating inflammation-driven cardiac remodeling.

### Potential mechanisms underlying ISO-mediated myocardial damage

**Figure 5:**
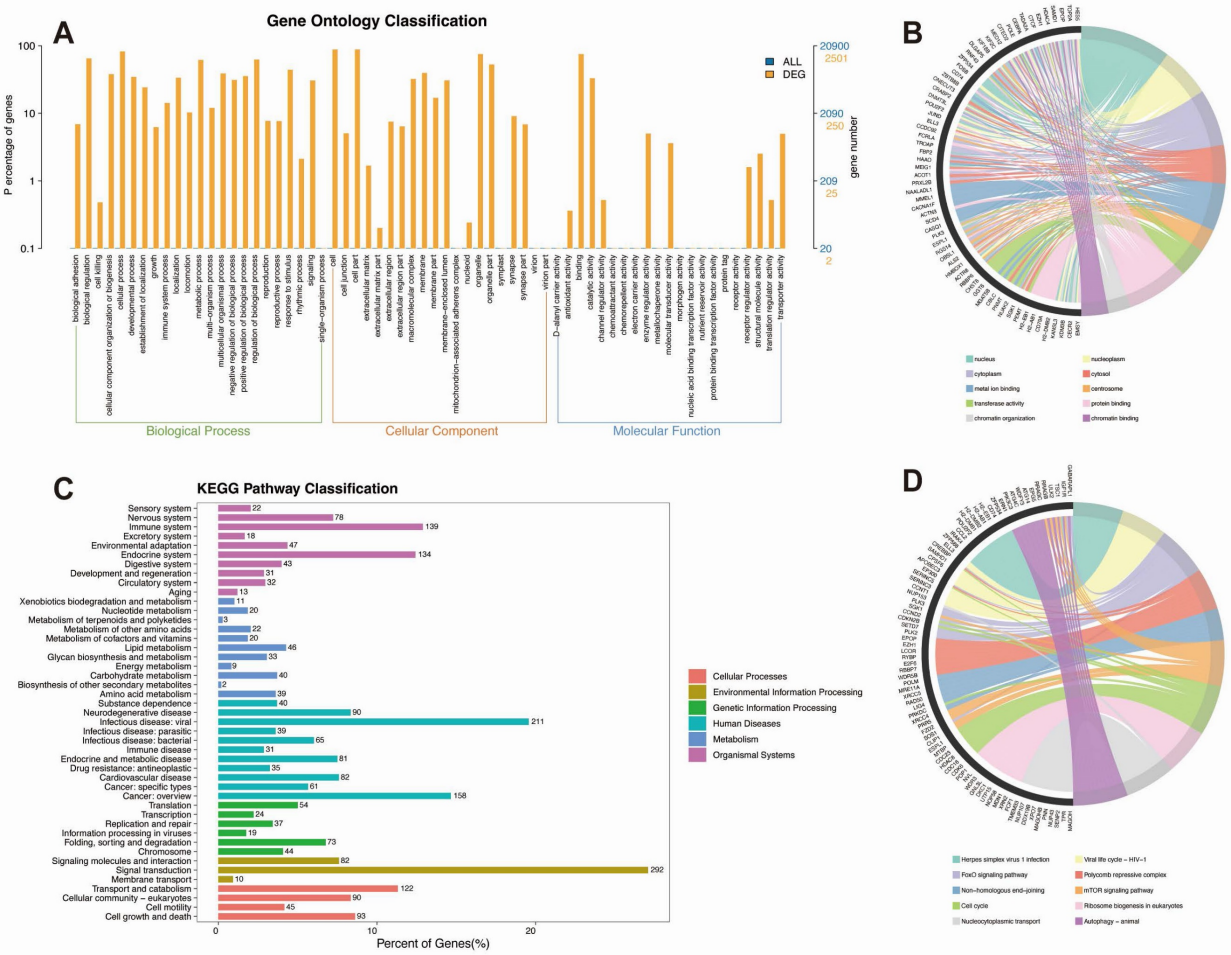
** GO and KEGG pathway enrichment analysis of differentially expressed genes (DEGs) in isoproterenol-treated mice. A.** GO analysis of DEGs. **B. C**hord diagram illustrating the connections between significantly enriched GO pathways and their associated genes.** C.** KEGG pathway classification of DEGs, showing the percentage of genes in each category compared to the entire set of genes. **D.** Chord diagram illustrating the connections between significantly enriched KEGG pathways and their associated genes.

To investigate the molecular mechanisms underlying isoproterenol-induced myocardial injury in mice, we conducted a comprehensive bioinformatics analysis using the GSE207581 dataset. The dataset included two experimental groups: mice receiving PBS (phosphate-buffered saline) injections (control group) and mice receiving isoproterenol injections (treatment group). GO enrichment analysis revealed significant enrichment of genes associated with various biological processes, cellular components, and molecular functions (Figure 5A-B). Notably, we observed enrichment of genes involved in DNA damage response, DNA repair, protein ubiquitination, and chromatin organization (Figure 5A-B). These processes are essential for maintaining genomic integrity and stability, suggesting that isoproterenol-induced myocardial injury may trigger DNA damage and activate repair mechanisms. Furthermore, the enrichment of genes related to the regulation of transcription by RNA polymerase II indicates significant transcriptional changes occurring in response to isoproterenol treatment (Figure 5A-B). Additionally, the involvement of cilium assembly and protein localization suggests potential alterations in cellular structures and functions, which may contribute to the development of myocardial injury.

KEGG pathway enrichment analysis identified several key pathways that were significantly enriched in the isoproterenol-treated group compared to the control group (Figure 5C-D). These pathways include autophagy, cell cycle, mTOR signaling, FoxO signaling, ubiquitin-mediated proteolysis, and nucleocytoplasmic transport. Autophagy is a cellular degradation process that plays a crucial role in maintaining cellular homeostasis and responding to stress (Figure 5C-D). The enrichment of autophagy-related genes suggests that isoproterenol may induce autophagy as a protective mechanism against myocardial injury (Figure 5C-D). The cell cycle pathway regulates cell growth and division, and its enrichment indicates that isoproterenol may affect cell proliferation in the heart. The mTOR and FoxO signaling pathways are involved in cell growth, survival, and metabolism, and their enrichment suggests that isoproterenol may modulate these signaling pathways to promote myocardial injury (Figure 5C-D). Ubiquitin-mediated proteolysis is a major protein degradation pathway, and its enrichment indicates that isoproterenol may induce protein degradation in the heart. Nucleocytoplasmic transport is essential for the proper localization of proteins and RNA molecules, and its enrichment suggests that isoproterenol may disrupt this process, leading to cellular dysfunction.

KEGG classification analysis revealed that the differentially expressed genes (DEGs) were enriched in various biological categories, including signal transduction, signaling molecules and interaction, cell growth and death, and cellular community - eukaryotes. The enrichment of genes related to signal transduction suggests that isoproterenol may activate multiple signaling pathways to induce myocardial injury (Figure 5C-D). The enrichment of genes related to signaling molecules and interaction indicates that isoproterenol may alter the expression and function of various signaling molecules, leading to dysregulation of cellular processes (Figure 5C-D). The enrichment of genes related to cell growth and death suggests that isoproterenol may affect cell proliferation and survival in the heart (Figure 5C-D). The enrichment of genes related to cellular community - eukaryotes indicates that isoproterenol may disrupt cellular interactions and communication, contributing to the development of myocardial injury.

Overall, our GO and KEGG analyses provide valuable insights into the molecular mechanisms and pathways involved in isoproterenol-induced myocardial injury in mice. The enrichment of genes and pathways related to DNA damage response, DNA repair, ubiquitination, chromatin organization, cell cycle, autophagy, and various signaling pathways suggests a complex interplay between these processes. These findings could have implications for understanding the pathogenesis of myocardial injury and identifying potential therapeutic targets for its treatment.

## Discussion

Our study demonstrates that the Mst1/Hippo signaling pathway plays a crucial role in isoproterenol-induced myocardial injury and subsequent cardiac remodeling. We observed that Mst1 ablation in cardiomyocytes protected against ISO-induced cardiac dysfunction, as evidenced by improved fractional shortening and preserved cardiomyocyte viability. This protective effect was attributed to the inhibition of cardiomyocyte apoptosis, oxidative stress, and calcium overload, all of which are key contributors to myocardial injury.

Mechanistically, our findings reveal that Mst1 deletion mitigates endoplasmic reticulum (ER) stress and mitochondrial fission, two critical processes implicated in ISO-mediated cardiac damage. ER stress, triggered by the accumulation of unfolded or misfolded proteins, can lead to cardiomyocyte apoptosis and dysfunction. Mitochondrial fission, the division of mitochondria into smaller fragments, is an early hallmark of mitochondrial dysfunction and has been linked to various cardiovascular diseases. By inhibiting these processes, Mst1 ablation effectively protects cardiomyocytes from ISO-induced injury.

Isoproterenol (ISO), a synthetic β-adrenergic receptor agonist, is a well-established model for inducing myocardial injury in experimental animals. Recent investigations have elucidated the underlying mechanisms of ISO-mediated cardiac damage, offering insights into the pathophysiology of myocardial injury and potential therapeutic interventions. Excessive activation of β-adrenergic receptors by ISO leads to intracellular calcium overload and oxidative stress 20, 21. This calcium dysregulation disrupts mitochondrial function, culminating in the opening of the mitochondrial permeability transition pore (mPTP) and the release of pro-apoptotic factors such as cytochrome c. This cascade triggers cardiomyocyte apoptosis, a major contributor to ISO-induced myocardial injury 22. Furthermore, ISO-mediated oxidative stress activates several signaling pathways, including the mitogen-activated protein kinase (MAPK) and nuclear factor-κB (NF-κB) pathways, resulting in the upregulation of pro-inflammatory cytokines like TNF-α and IL-6 23, 24. This inflammatory response exacerbates myocardial damage. Recent studies have also implicated impaired autophagy in ISO-induced myocardial injury. ISO administration disrupts autophagic flux, leading to the accumulation of damaged proteins and organelles, thereby contributing to cardiomyocyte dysfunction and death 25, 26. In summary, ISO-induced myocardial injury is a multifaceted process involving calcium overload, oxidative stress, apoptosis, inflammation, and dysfunctional autophagy.

Furthermore, our study highlights the pivotal role of Mst1 in modulating the inflammatory response following ISO treatment. We observed that Mst1 deletion suppressed the expression of pro-inflammatory cytokines and inhibited neutrophil infiltration into the myocardium. This suggests that Mst1 may be a key regulator of inflammation-induced cardiac remodeling.

Inflammation, triggered by myocardial injury or stress, initiates a cascade of cellular and molecular events that contribute to remodeling. Pro-inflammatory cytokines, such as TNF-α, IL-1β, and IL-6, released by activated immune cells and cardiomyocytes, stimulate matrix metalloproteinase (MMP) activity 27, 28. This results in extracellular matrix (ECM) degradation, leading to ventricular dilation and impaired systolic function 29. Furthermore, inflammation promotes the activation of cardiac fibroblasts, key mediators of collagen and other ECM component deposition 30, 31. This excessive accumulation of ECM (fibrosis) increases myocardial stiffness, impairing diastolic function. This fibrotic response is mediated by TGF-β, a pro-fibrotic cytokine upregulated during inflammation 32. Additionally, inflammation induces oxidative stress, exacerbating myocardial remodeling. Reactive oxygen species (ROS) generated during inflammation can directly damage cardiomyocytes and activate signaling pathways that promote hypertrophy and apoptosis 33. ROS can also modify ECM proteins, leading to increased cross-linking and stiffness. Recent studies have identified potential therapeutic targets to modulate inflammation and mitigate myocardial remodeling, including anti-inflammatory agents (e.g., IL-1 receptor antagonists, TNF-α inhibitors), antioxidants, and MMP inhibitors 34-37. However, further research is needed to translate these findings into clinical applications. In conclusion, inflammation is a critical factor in the pathogenesis of myocardial remodeling, contributing to ECM degradation, fibrosis, oxidative stress, and cardiomyocyte dysfunction. Understanding the complex interplay between inflammation and myocardial remodeling is essential for developing targeted therapies to prevent or reverse this deleterious process.

Mammalian sterile 20-like kinase 1 (Mst1), a serine/threonine kinase, has garnered significant attention in recent years for its pivotal role in myocardial injury 38-40. Extensive research has investigated the involvement of Mst1 in diverse cardiovascular diseases, positioning it as a potential therapeutic target. Mst1 is activated in response to various stressors, such as oxidative stress, ischemia/reperfusion injury, and pressure overload 41, 42. Upon activation, Mst1 phosphorylates and modulates downstream targets, including the Hippo pathway effector YAP (Yes-associated protein) 43. This phosphorylation leads to YAP cytoplasmic sequestration and degradation, inhibiting its pro-survival functions in cardiomyocytes 44. Moreover, Mst1 can directly induce cardiomyocyte apoptosis through multiple mechanisms. It phosphorylates and activates pro-apoptotic proteins such as Bcl-2-associated death promoter (BAD) and caspase-3, while also inhibiting the pro-survival Akt signaling pathway 45. These actions collectively promote cardiomyocyte death and exacerbate myocardial injury. Mst1 also plays a role in regulating autophagy, a cellular process critical for the degradation of damaged proteins and organelles 46, 47. Excessive Mst1 activation impairs autophagic flux, leading to the accumulation of dysfunctional mitochondria and increased oxidative stress in cardiomyocytes, thereby contributing to myocardial injury and heart failure. Recent studies have explored targeting Mst1 as a therapeutic strategy for myocardial injury. Genetic deletion or pharmacological inhibition of Mst1 has been shown to attenuate cardiomyocyte apoptosis, improve cardiac function, and reduce infarct size in animal models of myocardial infarction and pressure overload-induced heart failure 48, 49. In conclusion, Mst1 is a central mediator of myocardial injury, exerting its effects through the regulation of apoptosis, autophagy, and cell survival pathways. Targeting Mst1 represents a promising avenue for the development of novel therapies for cardiovascular diseases associated with myocardial injury. However, further research is needed to fully elucidate the complex mechanisms of Mst1 signaling and to develop safe and effective Mst1-targeted therapies. Our study reveals that Mst1 deletion effectively mitigates isoproterenol-induced myocardial injury by inhibiting cardiomyocyte apoptosis, oxidative stress, calcium overload, endoplasmic reticulum (ER) stress, mitochondrial fission, and inflammation. These findings are further corroborated by our bioinformatics analysis using the GSE207581 dataset.

The GO enrichment analysis of differentially expressed genes (DEGs) between isoproterenol-treated and control mice highlighted the activation of DNA damage response and repair pathways, as well as protein ubiquitination and chromatin organization processes. These findings suggest that isoproterenol induces DNA damage and activates repair mechanisms in cardiomyocytes, while also affecting protein turnover and chromatin structure. The enrichment of genes related to the regulation of transcription by RNA polymerase II indicates significant transcriptional changes occurring in response to isoproterenol, which may contribute to the observed cardiac dysfunction.

The KEGG pathway enrichment analysis identified several key pathways that were significantly enriched in the isoproterenol-treated group, including autophagy, cell cycle, mTOR signaling, FoxO signaling, ubiquitin-mediated proteolysis, and nucleocytoplasmic transport. These pathways are known to play crucial roles in cell survival, growth, proliferation, and response to stress. The enrichment of autophagy-related genes suggests that isoproterenol may induce autophagy as a protective mechanism against myocardial injury, while the enrichment of cell cycle-related genes indicates that isoproterenol may affect cell proliferation in the heart. The mTOR and FoxO signaling pathways are involved in cell growth, survival, and metabolism, and their enrichment suggests that isoproterenol may modulate these pathways to promote myocardial injury. The enrichment of ubiquitin-mediated proteolysis and nucleocytoplasmic transport pathways further emphasizes the dysregulation of protein turnover and cellular localization in response to isoproterenol.

Overall, our integrated experimental and bioinformatics analyses provide a comprehensive understanding of the molecular mechanisms underlying isoproterenol-induced myocardial injury. The identification of Mst1 as a key regulator of multiple pathological processes, including apoptosis, oxidative stress, ER stress, mitochondrial fission, and inflammation, highlights its potential as a therapeutic target for mitigating myocardial injury and adverse cardiac remodeling. Further investigation into the intricate crosstalk between Mst1 and these pathways may pave the way for the development of novel therapeutic strategies for myocardial infarction and heart failure.

In conclusion, our findings provide compelling evidence for the therapeutic potential of targeting the Mst1/Hippo signaling pathway in mitigating ISO-induced myocardial injury and adverse cardiac remodeling. By inhibiting cardiomyocyte apoptosis, oxidative stress, calcium overload, ER stress, mitochondrial fission, and inflammation, Mst1 ablation offers a multifaceted approach to protect the heart from ISO-induced damage. Further investigation into the precise mechanisms by which Mst1 regulates these processes may pave the way for the development of novel therapeutic strategies for myocardial infarction and heart failure.

## Supplementary Material

Supplementary figures and tables.

## Funding

This study is supported by the Zhongshan Social Public Welfare Science and Technology Research Project (2022B108).

## References

[1] Kossack M, Hein S, Juergensen L, Siragusa M, Benz A, Katus HA (2017). Induction of cardiac dysfunction in developing and adult zebrafish by chronic isoproterenol stimulation. J Mol Cell Cardiol.

[2] Zhang Q, Deng Y, Lai W, Guan X, Sun X, Han Q (2016). Maternal inflammation activated ROS-p38 MAPK predisposes offspring to heart damages caused by isoproterenol via augmenting ROS generation. Sci Rep.

[3] Sharma S, Iqubal A, Khan V, Sharma K, Najmi AK, Haque SE (2023). Icariin ameliorates oxidative stress-induced inflammation, apoptosis, and heart failure in isoproterenol-challenged Wistar rats. Iran J Basic Med Sci.

[4] Li JD, Cheng AY, Huo YL, Fan J, Zhang YP, Fang ZQ (2016). Bilateral Renal Denervation Ameliorates Isoproterenol-Induced Heart Failure through Downregulation of the Brain Renin-Angiotensin System and Inflammation in Rat. Oxid Med Cell Longev.

[5] Silvis MJM, Kaffka Genaamd Dengler SE, Odille CA, Mishra M, van der Kaaij NP, Doevendans PA (2020). Damage-Associated Molecular Patterns in Myocardial Infarction and Heart Transplantation: The Road to Translational Success. Front Immunol.

[6] Torp MK, Vaage J, Stensløkken KO (2023). Mitochondria-derived damage-associated molecular patterns and inflammation in the ischemic-reperfused heart. Acta Physiol (Oxf).

[7] Kesler A, Agrawal DK, Thankam FG (2022). Toll-like receptors and damage-associated molecular patterns in the pathogenesis of heart transplant rejection. Mol Cell Biochem.

[8] Algoet M, Janssens S, Himmelreich U, Gsell W, Pusovnik M, Van den Eynde J (2023). Myocardial ischemia-reperfusion injury and the influence of inflammation. Trends Cardiovasc Med.

[9] Carrillo-Salinas FJ, Ngwenyama N, Anastasiou M, Kaur K, Alcaide P (2019). Heart Inflammation: Immune Cell Roles and Roads to the Heart. Am J Pathol.

[10] Song X, Yuan B, Zhao S, Zhao D (2022). Effect of taurine on the proliferation, apoptosis and MST1/Hippo signaling in prostate cancer cells. Transl Cancer Res.

[11] Ma S, Meng Z, Chen R, Guan KL (2019). The Hippo Pathway: Biology and Pathophysiology. Annu Rev Biochem.

[12] Fu M, Hu Y, Lan T, Guan KL, Luo T, Luo M (2022). The Hippo signalling pathway and its implications in human health and diseases. Signal Transduct Target Ther.

[13] Heallen T, Zhang M, Wang J, Bonilla-Claudio M, Klysik E, Johnson RL (2011). Hippo pathway inhibits Wnt signaling to restrain cardiomyocyte proliferation and heart size. Science.

[14] Chen X, Li Y, Luo J, Hou N (2020). Molecular Mechanism of Hippo-YAP1/TAZ Pathway in Heart Development, Disease, and Regeneration. Front Physiol.

[15] Wu W, Ziemann M, Huynh K, She G, Pang ZD, Zhang Y (2021). Activation of Hippo signaling pathway mediates mitochondria dysfunction and dilated cardiomyopathy in mice. Theranostics.

[16] Shang X, Zhang Y, Xu J, Li M, Wang X, Yu R (2020). SRV2 promotes mitochondrial fission and Mst1-Drp1 signaling in LPS-induced septic cardiomyopathy. Aging (Albany NY).

[17] Liu M, Yan M, He J, Lv H, Chen Z, Peng L (2021). Macrophage MST1/2 Disruption Impairs Post-Infarction Cardiac Repair via LTB4. Circ Res.

[18] Cheng Z, Zhang M, Hu J, Lin J, Feng X, Wang S (2018). Mst1 knockout enhances cardiomyocyte autophagic flux to alleviate angiotensin II-induced cardiac injury independent of angiotensin II receptors. J Mol Cell Cardiol.

[19] Zhang M, Zhang L, Hu J, Lin J, Wang T, Duan Y (2016). MST1 coordinately regulates autophagy and apoptosis in diabetic cardiomyopathy in mice. Diabetologia.

[20] Hunter DR, Haworth RA, Berkoff HA (1983). Modulation of cellular calcium stores in the perfused rat heart by isoproterenol and ryanodine. Circ Res.

[21] Harding SE, Vescovo G, Kirby M, Jones SM, Gurden J, Poole-Wilson PA (1988). Contractile responses of isolated adult rat and rabbit cardiac myocytes to isoproterenol and calcium. J Mol Cell Cardiol.

[22] Zhuo XZ, Wu Y, Ni YJ, Liu JH, Gong M, Wang XH (2013). Isoproterenol instigates cardiomyocyte apoptosis and heart failure via AMPK inactivation-mediated endoplasmic reticulum stress. Apoptosis.

[23] Xu GR, Zhang C, Yang HX, Sun JH, Zhang Y, Yao TT (2020). Modified citrus pectin ameliorates myocardial fibrosis and inflammation via suppressing galectin-3 and TLR4/MyD88/NF-κB signaling pathway. Biomed Pharmacother.

[24] Sun H, Bai J, Sun Y, Zhen D, Fu D, Wang Y (2023). Oxymatrine attenuated isoproterenol-induced heart failure via the TLR4/NF-κB and MAPK pathways *in vivo* and *in vitro*. Eur J Pharmacol.

[25] Liu BY, Li L, Liu GL, Ding W, Chang WG, Xu T (2021). Baicalein attenuates cardiac hypertrophy in mice via suppressing oxidative stress and activating autophagy in cardiomyocytes. Acta Pharmacol Sin.

[26] Liao M, Xie Q, Zhao Y, Yang C, Lin C, Wang G (2022). Main active components of Si-Miao-Yong-An decoction (SMYAD) attenuate autophagy and apoptosis via the PDE5A-AKT and TLR4-NOX4 pathways in isoproterenol (ISO)-induced heart failure models. Pharmacol Res.

[27] Paulus WJ, Tschöpe C (2013). A novel paradigm for heart failure with preserved ejection fraction: comorbidities drive myocardial dysfunction and remodeling through coronary microvascular endothelial inflammation. J Am Coll Cardiol.

[28] Kologrivova I, Shtatolkina M, Suslova T, Ryabov V (2021). Cells of the Immune System in Cardiac Remodeling: Main Players in Resolution of Inflammation and Repair After Myocardial Infarction. Front Immunol.

[29] Bacmeister L, Schwarzl M, Warnke S, Stoffers B, Blankenberg S, Westermann D (2019). Inflammation and fibrosis in murine models of heart failure. Basic Res Cardiol.

[30] Maruyama K, Imanaka-Yoshida K (2022). The Pathogenesis of Cardiac Fibrosis: A Review of Recent Progress. Int J Mol Sci.

[31] Zhang Y, Chen W, Wang Y (2020). STING is an essential regulator of heart inflammation and fibrosis in mice with pathological cardiac hypertrophy via endoplasmic reticulum (ER) stress. Biomed Pharmacother.

[32] Khalil H, Kanisicak O, Prasad V, Correll RN, Fu X, Schips T (2017). Fibroblast-specific TGF-β-Smad2/3 signaling underlies cardiac fibrosis. J Clin Invest.

[33] Zhang X, Qu H, Yang T, Liu Q, Zhou H (2022). Astragaloside IV attenuate MI-induced myocardial fibrosis and cardiac remodeling by inhibiting ROS/caspase-1/GSDMD signaling pathway. Cell Cycle.

[34] Xie M, Burchfield JS, Hill JA (2013). Pathological ventricular remodeling: therapies: part 2 of 2. Circulation.

[35] Murphy SP, Kakkar R, McCarthy CP, Januzzi JL (2020). Inflammation in Heart Failure: JACC State-of-the-Art Review. Journal of the American College of Cardiology.

[36] Sun Y (2009). Myocardial repair/remodelling following infarction: roles of local factors. Cardiovasc Res.

[37] Opal SM, DePalo VA (2000). Anti-inflammatory cytokines. Chest.

[38] Wang S, Fan Y, Feng X, Sun C, Shi Z, Li T (2018). Nicorandil alleviates myocardial injury and post-infarction cardiac remodeling by inhibiting Mst1. Biochem Biophys Res Commun.

[39] Zhao HX, Zhang Z, Zhou HL, Hu F, Yu Y (2020). Exercise training suppresses Mst1 activation and attenuates myocardial dysfunction in mice with type 1 diabetes. Can J Physiol Pharmacol.

[40] Shang X, Li J, Yu R, Zhu P, Zhang Y, Xu J (2019). Sepsis-related myocardial injury is associated with Mst1 upregulation, mitochondrial dysfunction and the Drp1/F-actin signaling pathway. J Mol Histol.

[41] Maejima Y, Zablocki D, Nah J, Sadoshima J (2023). The role of the Hippo pathway in autophagy in the heart. Cardiovasc Res.

[42] Shao Y, Wang Y, Sun L, Zhou S, Xu J, Xing D (2023). MST1: A future novel target for cardiac diseases. Int J Biol Macromol.

[43] Yang Y, Wang H, Ma Z, Hu W, Sun D (2018). Understanding the role of mammalian sterile 20-like kinase 1 (MST1) in cardiovascular disorders. J Mol Cell Cardiol.

[44] Zhou J (2014). An emerging role for Hippo-YAP signaling in cardiovascular development. J Biomed Res.

[45] Del Re DP, Matsuda T, Zhai P, Maejima Y, Jain MR, Liu T (2014). Mst1 promotes cardiac myocyte apoptosis through phosphorylation and inhibition of Bcl-xL. Mol Cell.

[46] Maejima Y, Kyoi S, Zhai P, Liu T, Li H, Ivessa A (2013). Mst1 inhibits autophagy by promoting the interaction between Beclin1 and Bcl-2. Nat Med.

[47] MST1 Inhibits Autophagy and Promotes Apoptosis by Phosphorylating Beclin-1 Cancer Discovery. 2013; 3: OF10-OF.

[48] Cheng Z, Zhang M, Hu J, Lin J, Feng X, Wang S (2019). Cardiac-specific Mst1 deficiency inhibits ROS-mediated JNK signalling to alleviate Ang II-induced cardiomyocyte apoptosis. J Cell Mol Med.

[49] Liu M, Yan M, He J, Lv H, Chen Z, Peng L (2021). Macrophage MST1/2 Disruption Impairs Post-Infarction Cardiac Repair via LTB4. Circulation Research.

