# Supplementary material for: Mst1/Hippo signaling pathway drives isoproterenol-induced inflammatory heart remodeling
Source: Int J Med Sci. 2024 Jul 1;21(9):1718–29. doi: 10.7150/ijms.95850 (PMC11241096; doi:10.7150/ijms.95850)
Supplement: Supplementary file 1 — Supplementary figures and tables. [file ijmsv21p1718s1.pdf]

Supplementary Materials

List of Supplementary Materials
Figure S1 to S13
Table S1 to S2

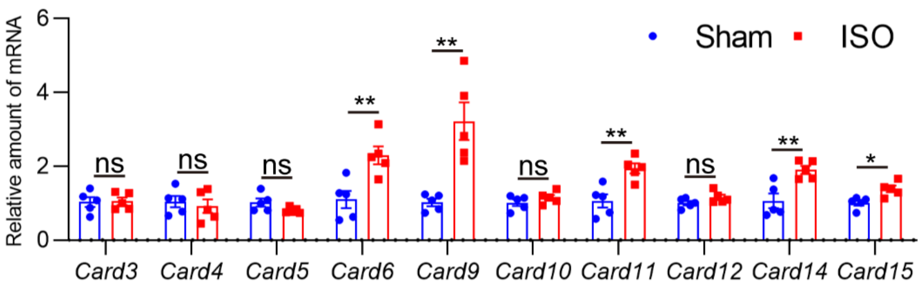

Supplementary Figure S1: The mRNA levels of *Card9* were increased in mouse hearts tissues following ISO infusion.

WT mice were administered 30 mg·kg^{-1}·d^{-1} ISO for two weeks. The mRNA levels of *Card* family genes in cardiac tissues. *Actb* mRNA was used as loading control ($n = 5$). Data were shown as mean ± SEM; **P* < 0.05; ***P* < 0.01; ns = not significant.

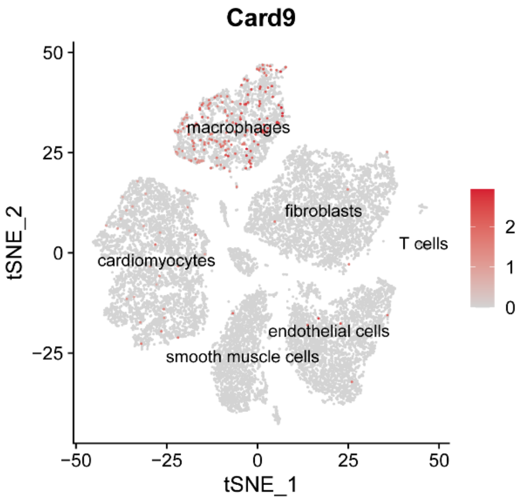

Supplementary Figure S2: The expression of *Card9* mRNA was predominantly localized within macrophages in the cardiac tissue.

The tSNE plot showed that the *Card9* mRNA expression in 6 main cell types, including macrophages, cardiomyocytes, fibroblasts, endothelial cells, smooth muscle cells, and T cells.

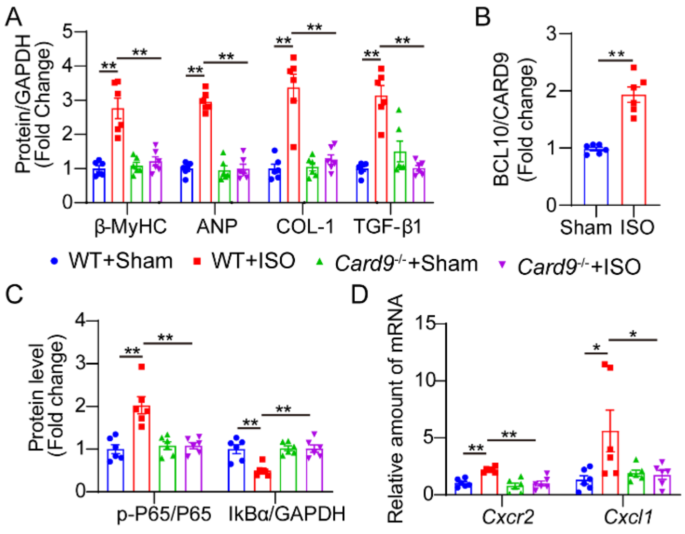

Supplementary Figure S3: CARD9 deficiency attenuated ISO-induced cardiac injury. *Card9*$^{-/-}$ mice and WT mice were administered 30 mg·kg^{-1}·d^{-1} ISO or an equal volume of sterile water for two weeks. (A) Densitometric quantification of immunoblots in Fig. 2G ($n = 6$). (B) Densitometric quantification of immunoblots in Fig. 2I ($n = 6$). (C) Densitometric quantification of immunoblots in Fig. 2J ($n = 6$). (D) The mRNA levels of *Cxcr2* and *Cxcl1* in heart tissues were determined using real-time PCR. *Actb* mRNA was used as a loading control ($n = 6$). Data were shown as mean ± SEM; *$P < 0.05$; **$P < 0.01$.

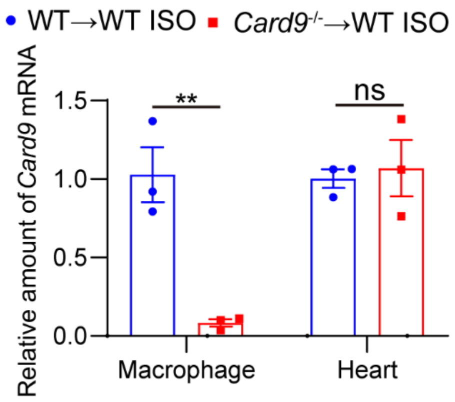

Supplementary Figure S4: Bone marrow transplant has been established successfully.
The mRNA levels of *Card9* in isolated macrophages and heart tissues were determined using real-time PCR. *Actb* mRNA was used as a loading control ($n = 3$). Data were shown as mean ± SEM; $^{**}P < 0.01$; ns=no significant.

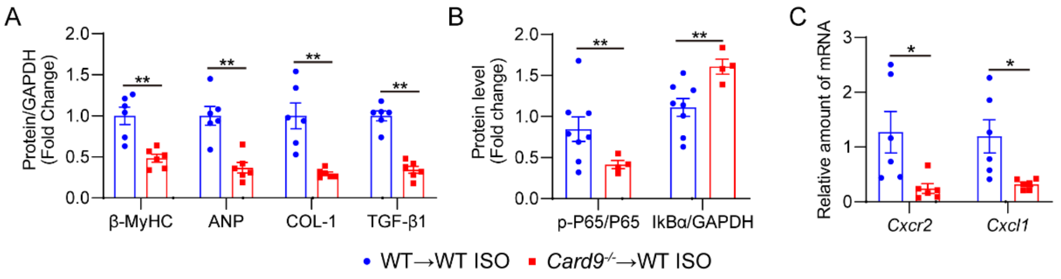

Supplementary Figure S5: Marrow-derived cell CARD9 mediated ISO-induced cardiac injuries.

WT mice were irradiated and administered bone marrow cells from either WT or *Card9*$^{-/-}$ mice. Mice were then administered 30 mg·kg^{-1}·d^{-1} ISO for 2 weeks. (A) Densitometric quantification of immunoblots in Fig. 3G (*n* =6). (B) Densitometric quantification of immunoblots in Fig. 3I (*n* =6). (C) The mRNA levels of *Cxcr2* and *Cxcl1* in heart tissues were determined using real-time PCR. *Actb* mRNA was used as a loading control (*n* = 6). Data were shown as mean ± SEM; *$P < 0.05$; **$P < 0.01$.

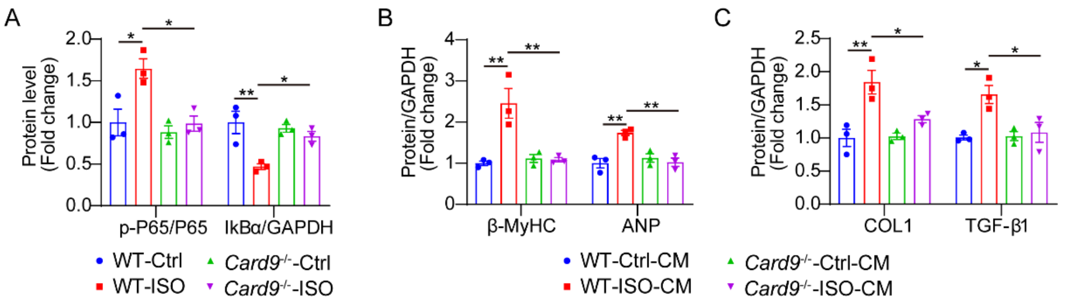

Supplementary Figure S6: Macrophage CARD9 blockade prevented ISO-induced inflammatory responses and then alleviated remodeling changes in cardiomyocytes and fibroblasts in vitro.

(A) Densitometric quantification of immunoblots in Fig. 4B (*n* =3). (B) Densitometric quantification of immunoblots in Fig. 4H (*n* =3). (C) Densitometric quantification of immunoblots in Fig. 4I (*n* =3). Data were shown as mean ± SEM; **P* < 0.05; ***P* < 0.01.

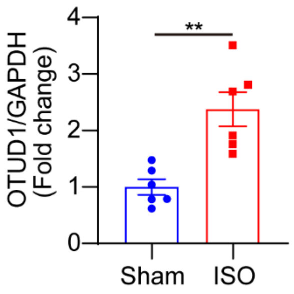

Supplementary Figure S7: The expression of OTUD1 was up-regulated in heart tissues upon ISO infusion.

Densitometric quantification of immunoblots in Fig. 5C (n =6). Data were shown as mean ± SEM; **$P < 0.01$.

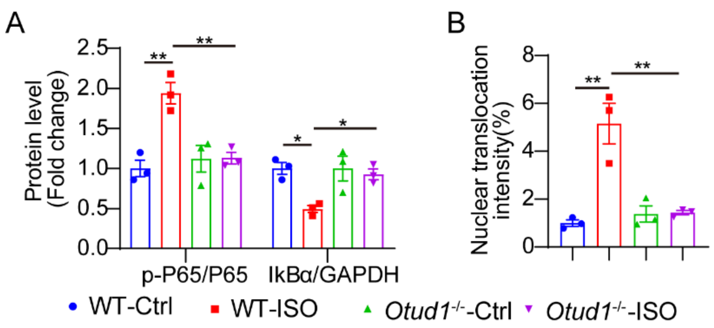

Supplementary Figure S8: OTUD1 deficiency inhibited ISO–induced inflammatory responses in macrophages.

(A) Densitometric quantification of immunoblots in Fig. 6C (*n* =3). (B) Quantification of intensity in Fig. 6D (*n* =3). Data were shown as mean ± SEM; **P* < 0.05; ***P* < 0.01.

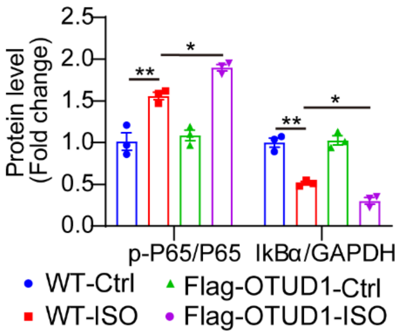

Supplementary Figure S9: OTUD1 overexpression accelerated ISO–induced inflammatory responses in macrophages.

Densitometric quantification of immunoblots in Fig. 6G (n =3). Data were shown as mean ± SEM; n = 3; * $P < 0.05$, ** $P < 0.01$.

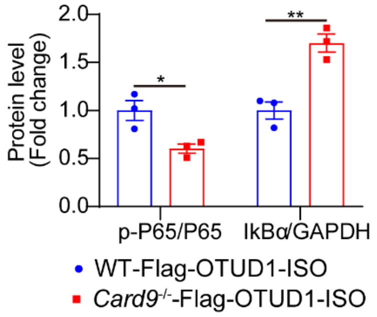

Supplementary Figure S10: NF-κB activation induced by OTUD1 overexpression in MPMs was significantly limited in *Card9* knockout mice.

Densitometric quantification of immunoblots in Fig. 6H (n =3). Data were shown as mean ± SEM; * $P < 0.05$, ** $P < 0.01$.

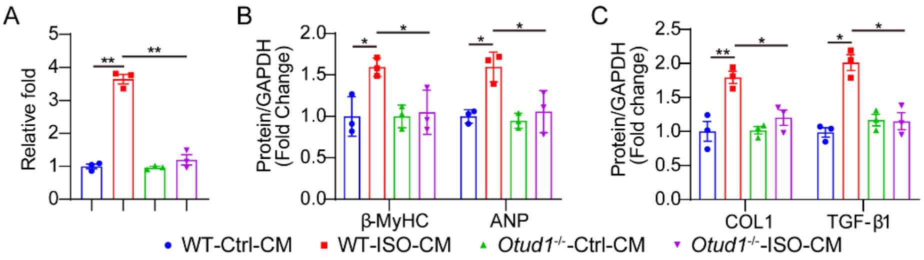

Supplementary Figure S11: Macrophage OTUD1 deficiency suppressed the cellular crosstalk between macrophages and other cardiac cells.

(A) Quantification of cell size in Fig. 6J. (B) Densitometric quantification of immunoblots in Fig. 6K (*n* =3). (C) Densitometric quantification of immunoblots in Fig. 6L (*n* =3). Data were shown as mean ± SEM; **P* < 0.05; ***P* < 0.01.

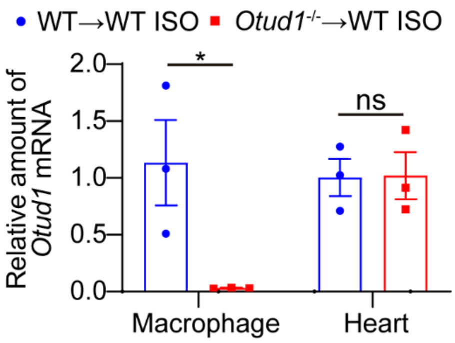

Supplementary Figure S12: Bone marrow transplant has been established successfully.

The mRNA levels of *Otud1* in isolated macrophages and heart tissues were determined using real-time PCR. *Actb* mRNA was used as a loading control (n =3). Data were shown as mean ± SEM; *P < 0.05; ns=no significant.

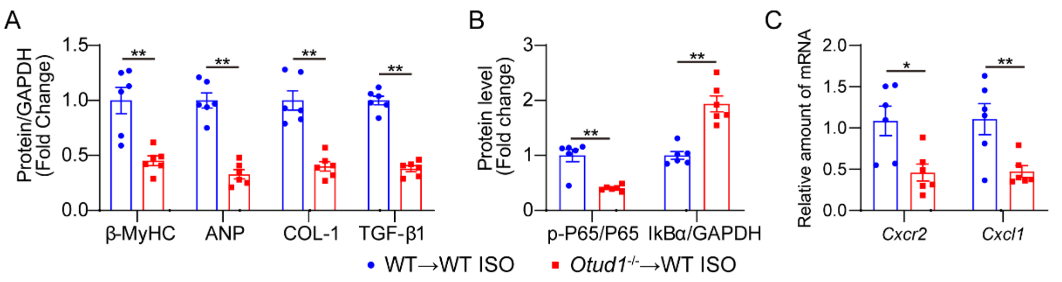

Supplementary Figure S13: Bone marrow-derived macrophage OTUD1 deficiency decreased ISO-induced cardiac remodeling.

WT mice were irradiated and administered bone marrow cells from either WT or *Otud1*$^{-/-}$ mice. Mice were then administered 30 mg·kg^{-1}·d^{-1} ISO for 2 weeks. (A) Densitometric quantification of immunoblots in Fig. 7I (n =6). (B) Densitometric quantification of immunoblots in Fig. 7K (n =6). (C) The mRNA levels of *Cxcr2* and *Cxcl1* in heart tissues were determined by real-time PCR. *Actb* mRNA was used as a loading control (n =6). Data were shown as mean ± SEM; *$P < 0.05$; **$P < 0.01$.

Supplementary Table S1. Reagent list used in this study.

Reagent	Source	Catalogue
DAPI	Beyotime Biotech	C1006
Rhodamine phalloidin	Abcam	ab176756
Lipofectamine 3000	Thermo Fisher	L3000015
Isoprenaline	Med Chem Express	HY-B0468
MG132	Med Chem Express	HY-13259
GAPDH antibody	Santa Cruz Biotechnology	sc-365062
CARD9 antibody	Santa Cruz Biotechnology	sc-374569
ANP antibody	Santa Cruz Biotechnology	sc-515701
Goat anti-mouse IgG H&L (TRITC) antibody	Abcam	ab6786
Goat anti-rabbit IgG H&L (Alexa Fluor 488) antibody	Abcam	ab150077
F4/80 antibody	Cell Signaling Technology	30325
phospho-NFκB P65 (Ser536) antibody	Cell Signaling Technology	3033
NF-κB P65 antibody	Cell Signaling Technology	8242
BCL10	Cell Signaling Technology	4237
HRP-conjugated goat anti-mouse IgG antibody	Cell Signaling Technology	7076
HRP-conjugated goat anti-rabbit IgG antibody	Cell Signaling Technology	7074
α-actinin antibody	Proteintech	11313-2-AP
Vimentin antibody	Proteintech	10366-1-AP
β-MyHC antibody	Proteintech	22280-1-AP
COL-1 antibody	Proteintech	14695-1-AP
TGF-β1 antibody	Proteintech	21898-1-AP
Myc tag antibody	Proteintech	16286-1-AP
DYKDDDDK tag antibody	Proteintech	66008-4-Ig
HA tag antibody	Proteintech	51064-2-AP
GFP tag antibody	Proteintech	50430-2-AP
FITC-conjugated wheat-germ agglutinin (WGA-FTIC)	Gene Tex	GTX01502
OTUD1 antibody	Bioss	bs-17563R
H&E kit	Solarbio Life Sciences	G1120
Picro Sirius Red stain	Solarbio Life Sciences	S8060
IL-6 mouse uncoated ELISA Kit	ebioscience	88-7064-77
TNF-α mouse uncoated ELISA Kit	ebioscience	88-7324-88

Supplementary Table S2. Primer sequences for qPCR.

Gene	Species	Squence
Card3	Mouse	AAATCATCCCCCACAGGAG<br>GGTCCAGGAGAACCAGTGTT
Card4	Mouse	TTTAAGGGTGAAGCCAAAGG<br>GGCAGACAAATCAGGATTCAG
Card5	Mouse	GAGCAGCTGCAAACGACTAA<br>GTCCACAAAGTGTCCTGTTCTG
Card6	Mouse	TTTCCTCCGGTGTTTGTCTAATG<br>GTTCACCCCCACAGTCTCTTC
Card9	Mouse	CTCTGTGCAGGAGGGTAAGC<br>TCCGTAGGGAGAAGATGGTG
Card10	Mouse	TGCAGGGCGAGCTACAGT<br>GCAGATCCTCCATCTCTTGC
Card11	Mouse	TCTCCAGAGCGAGTTTCTTCTT<br>TGTTTTCTGACCGGCTGAC
Card12	Mouse	TGATCTCCAAGAGATGAAGTTGG<br>GATCAAATTGTGAAGATTCTGTGC
Card14	Mouse	GAGAAACTCCGCTCCATGAC<br>CCTCATCCAGACTCTGTTCCA
Card15	Mouse	TGTGGAGTCACCGCAAAAC<br>TCCTCTGTGCCTGGAACTCT
Myh7	Mouse	CAAAGGCAAGGCAAAGAAAG<br>TCACCCCTGGAGACTTTGTC
Nppa	Mouse	AAGAACCTGCTAGACCACCTGGAG<br>TGCTTCCTCAGTCTGCTCACTCAG
Col1a1	Mouse	TGGCCTTGGAGGAAACTTTG<br>CTTGGAAACCTTGTGGACCAG
Tgfb1	Mouse	TGGAGCAACATGTGGAACTC<br>GTCAGCAGCCGGTTACCA
Il6	Mouse	GAGGATACCACTCCCAACAGACC<br>AAGTGCATCATCGTTGTTCATACA
Tnf	Mouse	TGATCCGCGACGTGGAA<br>ACCGCCTGGAGTTCTGGAA
Cxcr2	Mouse	ATGCCCTCTATTCTGCCAGAT<br>GTGCTCCGGTTGTATAAGATGAC
Cxcl1	Mouse	CTGGGATTCACCTCAAGAACATC<br>CAGGGTCAAGGCAAGCCTC
Clec7a	Mouse	GGGTGCCCTAGGAGGTTTTT<br>TGCTGATCCATCCTCCCAGA
Clec4n	Mouse	ATTTCATCACCCAGCAGC<br>AAAACATCATTCCAGCCCC
Clec4e	Mouse	ACACAGAGAGAGGATGCTTC<br>CTTGACTGAACCTGATGCC
Otud1	Mouse	AGAGGCAGGACAAGTACCTGA<br>CCCGTACACAGTCTTGCTGAC
Usp15	Mouse	CCGTGGATGAAAACCTGAGTAG<br>TTCTCTTAGGCAGACAGGGATAA
Trim62	Mouse	TGCGAGCACTACTTCTGCC<br>CTTGACCTTGTCGTGAGCC
Actb	Mouse	CCGTGAAAAGATGACCCAGA<br>TACGACCAGAGGCATACAG
